# Cutaneous leishmaniasis in a renal transplant patient: Diagnostic challenges in a non-endemic setting

**DOI:** 10.1016/j.idcr.2025.e02333

**Published:** 2025-07-27

**Authors:** Zishan Nasir, Bushra M. Abdallah, Mohamed Aboukamar, Syed Hidayat Ali, Saifatullah Khan, Sulieman Abujarir, Muna Almaslamani, Muftah Othman

**Affiliations:** a Division of Nephrology, Department of Medicine, Hazm Mebaireek General Hospital, Hamad Medical Corporation, Doha, Qatar; b College of Medicine, QU Health, Qatar University, Doha, Qatar; c Department of Medical Education, Hamad Medical Corporation, Doha, Qatar; d Infectious Diseases Division, Department of Medicine, Communicable Diseases Center, Hamad Medical Corporation, Doha, Qatar

**Keywords:** Leishmaniasis, Cutaneous Leishmaniasis, Renal transplant, Amphotericin B

## Abstract

Leishmanial infections, though uncommon in Southeast and Central Asia, remain clinically significant due to their potential to cause substantial morbidity and mortality. This case report presents a young Nepalese male, four years post-renal transplant and with chronic allograft dysfunction secondary to non-compliance, who presented with a right chest skin lesion and fever. Initially suspected to be cutaneous tuberculosis or malignancy, investigations, including a skin biopsy, revealed cutaneous leishmaniasis. The patient responded well to treatment with liposomal amphotericin B, highlighting the importance of considering leishmaniasis in differential diagnoses, especially in immunocompromised individuals from, or travelling to, endemic regions.

## Introduction

Leishmaniasis is a significant yet neglected tropical disease associated with substantial morbidity and mortality [1]. It is caused by protozoan parasites of the genus *Leishmania*, comprising over 20 different species. Transmission occurs via the bite of an infected female sandfly, with nearly 70 animal species, including humans, serving as potential reservoirs for the parasite [2]. Leishmanial infections manifest in three primary forms: visceral leishmaniasis (VL), cutaneous leishmaniasis (CL), and mucocutaneous leishmaniasis (MCL).

The *Leishmania* parasite has two distinct forms in its life cycle: the promastigote, which develops and resides extracellularly within the sandfly vector, and the amastigote, which multiplies intracellularly in the host's reticuloendothelial cells. In endemic regions, humans are the primary reservoir, but mammals such as rodents, dogs, and foxes have also been identified as potential reservoirs [3].

Cutaneous leishmaniasis can be misdiagnosed as other conditions, such as skin tuberculosis or fungal infections. Due to increased international travel and expatriate work, there is a growing number of sporadic cases of cutaneous leishmaniasis in non-endemic areas, warranting careful consideration. This case report presents the first documented case of cutaneous leishmaniasis in a post-kidney transplant patient in Qatar.

## Case report

A 44-year-old Nepalese male residing in Qatar, with a history of hypertension and IgA nephropathy leading to stage 4 chronic kidney disease, underwent a living-donor kidney transplant from his mother in Nepal four years before presentation (post-transplant day 0). Following the transplant, he developed delayed graft dysfunction with persistently elevated serum creatinine. His maintenance immunosuppressive therapy included mycophenolate mofetil, tacrolimus, and prednisolone. He also developed post-transplant diabetes mellitus (PTDM) managed with oral antidiabetics.

Approximately nine months ago (post-transplant 3 years), non-adherence to medications and clinic follow-up led to worsening renal function. A renal graft biopsy revealed acute T-cell-mediated rejection (Banff type IIA), treated with intravenous steroid pulse and intensified immunosuppression. While serum creatinine improved significantly, subsequent challenges three months later (post-transplant 3.5 years) included a urinary tract infection and poorly controlled blood sugars, which were managed with antibiotics and prednisolone tapering.

During a two-month vacation in Nepal, he reported an episode of chicken pox, evidenced by post-inflammatory hyperpigmentation on both legs, although no medical records were available. During his stay, he was non-compliant with his prescribed treatment. About 25 days before presenting to the emergency department (post-transplant 4 years), he developed a skin lesion on the upper right chest, initially appearing as a small pustule that gradually enlarged and ulcerated, producing scant purulent discharge. He denied any history of trauma to the site. He presented to the emergency department with complaints of the skin lesion, high-grade continuous fever for three days, chills, and rigors. He denied urinary symptoms, cough, chest pain, loose stools, or generalised swelling.

**Fig. 1:**
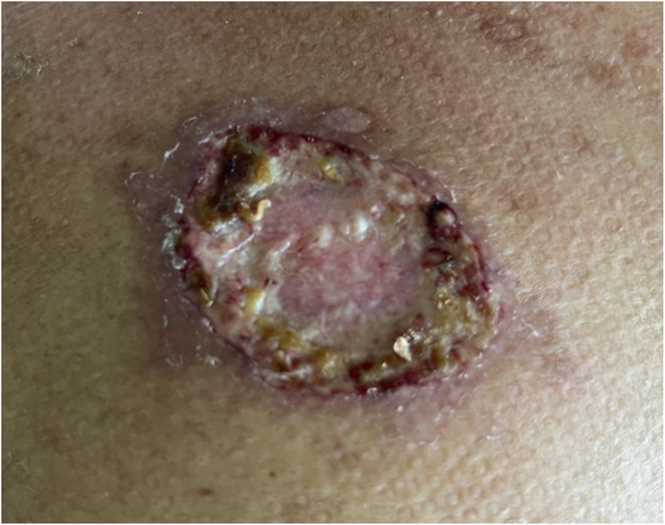
Clinical image of the cutaneous lesion at presentation. A 2 × 2 cm ulcer is seen on the upper right chest wall, just below the clavicle. The lesion displays red granulation tissue with purulent material at the edges, consistent with an evolving infectious skin ulcer in an immunosuppressed kidney transplant recipient.

On examination, he was febrile but otherwise haemodynamically stable. A 2 × 2 cm ulcer was observed on the upper half of the right chest wall, just below the right clavicle, displaying red granulation tissue with purulent material at the edges (Fig. 1).

Investigations showed elevated serum creatinine (500 μmol/L) and urea (18.4 mmol/L), along with severe anaemia (haemoglobin 7.2 g/dL), leucocytosis (white cell count 25,000/μL), thrombocytopenia (17,000/μL), and metabolic acidosis (serum bicarbonate 12 mmol/L). His CRP was elevated at 120 mg/L, and trough tacrolimus levels were suboptimal at 6 ng/mL. Imaging, including ultrasound of the renal graft, echocardiogram, and chest X-ray, was unremarkable.

He was initiated on broad-spectrum intravenous antibiotics, and blood and urine cultures were collected but were subsequently negative. Intravenous fluids were administered to correct metabolic acidosis and maintain hydration. Prednisolone was titrated up to 10 mg daily, mycophenolate was discontinued, and the tacrolimus dose was adjusted to 3 mg twice daily, guided by serial trough levels. Immunological tests, including antinuclear antibody (ANA), antineutrophil cytoplasmic antibody (ANCA), and anti-glomerular basement membrane (anti-GBM) antibody, were negative, and serum complement levels were within normal ranges. Infectious disease workup, including tests for tuberculosis, Brucella antibodies, and viruses such as herpes zoster, herpes simplex, cytomegalovirus, mumps virus, and SARS-CoV-2, also yielded negative results.

**Fig. 2:**
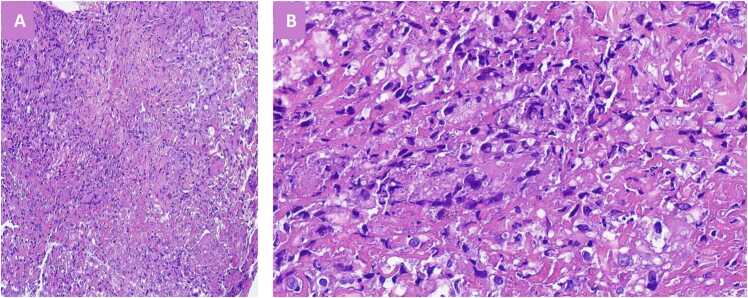
Histopathological findings from the skin punch biopsy. (A) Section showing caseating granulomatous inflammation in the dermis, consistent with a granulomatous infectious process. (B) Higher magnification of the same biopsy highlighting numerous Leishmania amastigotes appearing as small, uniform, round intracytoplasmic organisms within histiocytes, confirming the diagnosis of cutaneous leishmaniasis.

Despite treatment, he continued spiking fever, prompting a dermatologist to perform a punch biopsy of the lesion and recommend topical fusidic acid application. The biopsy revealed superficial skin fragments with focal ulceration, caseating granulomatous inflammation in the dermis, and numerous *Leishmania* particles within histiocytes (Fig. 2). There was no evidence of fungi, atypia, or malignancy.

All antibiotics were discontinued, and intravenous liposomal amphotericin B (200 mg; 3 mg/kg) was administered daily for five days, with additional outpatient doses scheduled on days 14 and 21 after the first amphotericin B dose. The ulcer was dressed daily with gentamicin ointment. Renal function was closely monitored through daily laboratory investigations to ensure treatment safety in the context of his existing renal impairment.

**Fig. 3:**
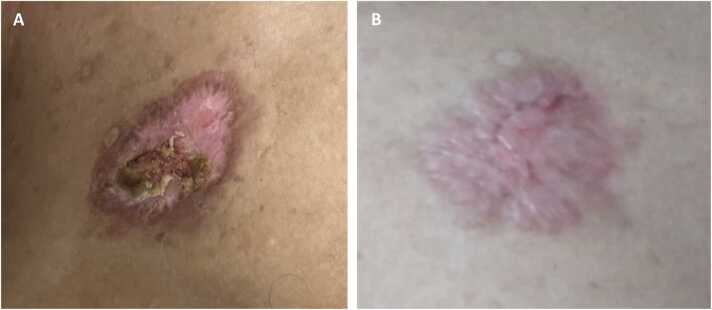
Clinical progression of the skin lesion following treatment with amphotericin B. (A) Image taken two weeks after initiation of therapy shows marked reduction in erythema and granulation tissue. (B) Image at six weeks demonstrates near-complete healing with re-epithelialisation and resolution of purulence, indicating good clinical response to treatment.

**Fig. 4:**
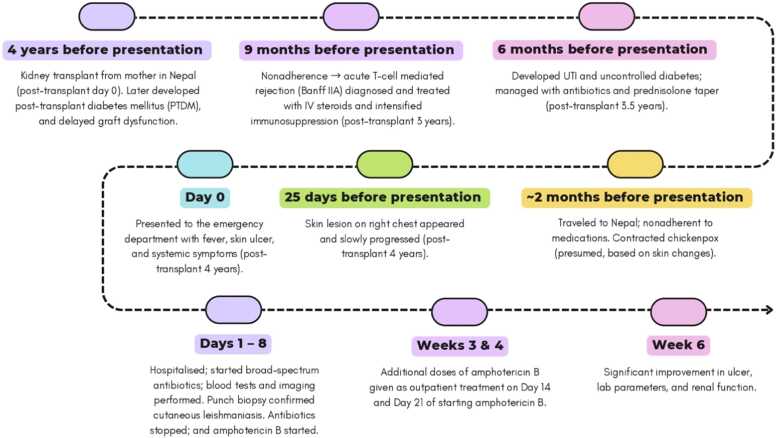
Timeline summarising key clinical events. This schematic outlines the chronological sequence of major clinical events, including the patient's renal transplant, diagnosis, and treatment of cutaneous leishmaniasis, using presentation to the emergency department with the skin ulcer as the reference point (Day 0). Abbreviations: IV, intravenous; UTI, urinary tract infection; lab, laboratory.

His skin lesion showed significant improvement with the treatment (Fig. 3). Renal function improved, haemoglobin levels increased, and leucocytosis and thrombocytopenia resolved, with CRP levels returning to normal. Mycophenolate was resumed at a reduced dose of 250 mg twice daily upon discharge. The patient was advised to adhere strictly to his treatment regimen and attend regular follow-up appointments in the outpatient clinic. Fig. 4 presents a visual timeline of the clinical events for the patient’s presentation.

## Discussion

Leishmaniasis is a vectorborne disease caused by protozoan parasites from the genus *Leishmania*. It is endemic in large regions of the tropics, subtropics, and the Mediterranean basin, affecting over 98 countries [4]. The disease manifests in three primary forms: visceral leishmaniasis (VL), cutaneous leishmaniasis (CL), and mucocutaneous leishmaniasis (MCL) [2], [5].

Cutaneous leishmaniasis is the most prevalent form and presents with skin lesions that may leave lifelong scars, resulting in disability and social stigma. Almost 95 % of CL cases occur in the Americas, Central Asia, the Middle East, and the Mediterranean, with an estimated 600,000–1 million new cases annually, although only 200,000 are reported to the World Health Organisation (WHO) [2]. There are two types of CL: localised and diffuse, the latter being difficult to treat [6]. In immunocompromised patients, such as transplant recipients, CL may manifest atypically, mimic malignancy or pyogenic abscess, or progress to more severe and disseminated forms [5], [7], [8].

Key risk factors for leishmaniasis include poverty, poor sanitation, malnutrition, and environmental changes like urbanisation and deforestation. Climate change and natural disasters exacerbate the disease's spread by influencing sandfly populations and human migration [2]. Immunosuppression, whether due to HIV or non-HIV conditions such as organ transplantation and autoimmune disorders, significantly increases susceptibility [5]. Transplant recipients are particularly at risk, as immunosuppressive therapies compromise T-cell function, making them vulnerable to infections [7]. In rheumatology, leishmaniasis has also been linked to the use of immunosuppressive drugs, including azathioprine, methotrexate, steroids, cyclosporine, and cyclophosphamide [9].

Our patient, a kidney transplant recipient on long-term immunosuppression, presented with a solitary ulcerated lesion suspicious for malignancy, which was later confirmed to be CL. This clinical presentation mirrors that described by Akay et al., who reported a post-liver transplant patient with a CL lesion mimicking basosquamous carcinoma while on tacrolimus and mycophenolate mofetil [10]. Amiri et al. described a disseminated CL case in a kidney transplant recipient who initially responded to oral miltefosine but relapsed and later required amphotericin B [11]. However, in contrast to our case, their patient suffered graft function deterioration and became dialysis-dependent. Our patient, despite presenting with fever and pancytopenia in addition to a skin ulcer, had only localised disease and responded rapidly to liposomal amphotericin B without compromising graft function.

Antinori et al. conducted a comprehensive review of leishmaniasis in organ transplant recipients, identifying 79 cases by 2008; a four-fold increase since the early 1990s [8]. Notably, 77 % of cases were diagnosed following renal transplantation, which is the most commonly performed transplant procedure. Visceral leishmaniasis accounted for 86 % of cases, while mucocutaneous leishmaniasis was less common, and cutaneous leishmaniasis was rarely observed [8]. Leishmaniasis in transplant recipients, often presenting as urinary abnormalities or acute kidney injury (AKI), is associated with increased mortality [12], [13]. Renal damage can result from immune mechanisms like immune complex deposition and inflammatory responses or direct parasite proliferation within the kidneys [14].

A recent local study by Abukhattab et al. identified 68 cases of leishmaniasis in Qatar from 2016 to 2022, of which 88.2 % were CL, 10.3 % VL, and 1.5 % MCL [15]. Skin lesions were the predominant presentation (79.4 %), further emphasising CL’s relevance in this region.

Diagnostic tools, like *Leishmania* polymerase chain reaction (PCR), offer high sensitivity and can be used to monitor disease activity and treatment response [8]. Standard treatments for CL involve antimonial drugs, but these can have significant toxicity. Liposomal amphotericin B is effective for VL and CL, offering lower toxicity compared to antimonials, despite potential side effects like nephrotoxicity and anaemia [7], [8], [16]. Alternatives like ketoconazole and itraconazole have also shown efficacy [7], [17]. Liposomal amphotericin B was selected for treatment in our case due to its favourable safety profile and prior use in our facility. The patient's renal function was closely monitored with daily laboratory tests, allowing for early detection of any deterioration. Fortunately, he tolerated the treatment well.

Given the increased global travel and advanced medical interventions, leishmaniasis among immunocompromised individuals is likely to rise, necessitating heightened awareness and improved surveillance systems to address this emerging concern. Cutaneous manifestations in such populations should prompt consideration of leishmaniasis, especially in endemic or travel-related contexts.

## Conclusion

In conclusion, this case highlights the complexity of managing *Leishmania* infections in immunosuppressed individuals, particularly in post-renal transplant patients. Immunosuppressive therapy, while crucial for transplant survival, increases susceptibility to opportunistic infections such as leishmaniasis, which may present atypically and complicate both diagnosis and treatment. Prompt recognition through histopathological examination and targeted treatment with liposomal amphotericin B can result in significant clinical improvement. This case emphasises the need for heightened awareness, early diagnostic measures, and tailored therapeutic approaches in immunocompromised populations, especially in endemic regions or among travellers returning from such areas.

## Author Statement

All authors made a significant contribution to the work reported; took part in drafting, revising or critically reviewing the manuscript; gave final approval of the current version; and agreed to be accountable for all aspects of the work.

## CRediT authorship contribution statement

**Zishan Nasir:** Writing – review & editing, Writing – original draft, Project administration, Conceptualization. **Bushra M Abdallah:** Writing – review & editing, Writing – original draft, Visualization, Validation, Project administration, Conceptualization. **Muftah Othman:** Writing – review & editing, Writing – original draft, Supervision, Project administration, Conceptualization. **Sulieman Abujarir:** Writing – review & editing, Validation, Conceptualization. **Muna Almaslamani:** Writing – review & editing, Validation, Conceptualization. **Syed Hidayat Ali:** Writing – review & editing, Writing – original draft, Methodology, Conceptualization. **Saifatullah Khan:** Writing – review & editing, Writing – original draft, Methodology, Conceptualization. **Mohamed Aboukamar:** Writing – review & editing, Writing – original draft, Visualization, Conceptualization.

## Ethical approval

The case report received ethical approval from our institution with approval number (MRC-04-24-844).

## Ethics

Patient’s consent was taken for scientific publications. The case report received ethical approval from our institution with approval number (MRC-04-24-844).

## Consent

Written informed consent was obtained from the patient for publication of this case report and accompanying images. A copy of the written consent is available for review by the Editor-in-Chief of this journal on request.

## Funding

This work did not receive any specific grant from funding agencies in the public, commercial or not-for-profit sectors.

## Disclosures

The authors report no conflicts of interest in this work.

## Declaration of Competing Interest

The authors declare that they have no known competing financial interests or personal relationships that could have appeared to influence the work reported in this paper.

## References

[1] Joudeh A.I., Elsiddig Awadelkarim H.A., Gul M.I., Elayana M.S., Soliman D.S., Amer A. (2023). Visceral leishmaniasis complicated by hemophagocytic lymphohistiocytosis: a case report from a nonendemic area. Clin Case Rep.

[2] Leishmaniasis. World Health Organization; 2023 [Available from: 〈https://www.who.int/news-room/fact-sheets/detail/leishmaniasis#:~:text=Leishmaniasis%20is%20caused%20by%20protozoan,and%20lack%20of%20financial%20resources〉].

[3] Gawade S., Nanaware M., Gokhale R., Adhav P. (2012). Visceral leishmaniasis: a case report. Austral Med J.

[4] Akhoundi M., Kuhls K., Cannet A., Votýpka J., Marty P., Delaunay P. (2016). A historical overview of the classification, evolution, and dispersion of leishmania parasites and sandflies. PLoS Negl Trop Dis.

[5] van Griensven J., Carrillo E., López-Vélez R., Lynen L., Moreno J. (2014). Leishmaniasis in immunosuppressed individuals. Clin Microbiol Infect.

[6] Goto H., Lindoso J.A. (2010). Current diagnosis and treatment of cutaneous and mucocutaneous leishmaniasis. Expert Rev Anti Infect Ther.

[7] Yaich S., Charfeddine K., Masmoudi A., Masmoudi M., Zaghdhane S., Turki H. (2013). Atypical presentation of cutaneous leishmaniasis in a renal transplant recipient successfully treated with allopurinol and fluconazole. Ann Saudi Med.

[8] Antinori S., Cascio A., Parravicini C., Bianchi R., Corbellino M. (2008). Leishmaniasis among organ transplant recipients. Lancet Infect Dis.

[9] Viana R.B., Neiva C.L., Dias A.F., do Rosário e Souza E.J., de Pádua P.M. (2010). Felty's syndrome and Kala-azar: a challenge for the rheumatologist. Rev Bras Reumatol.

[10] Akay B.N., Atak M.F., Okcu Heper A., Farabi B. (2020). Cutaneous leishmaniasis dermatoscopically mimicking basosquamous carcinoma in a solid organ transplant recipient. Dermatol Ther.

[11] Amiri R., Farrokhnia M., Mousavi Mehdiabadi F. (2023). Disseminated cutaneous leishmaniasis in a kidney transplant recipient. Clin Case Rep.

[12] Clementi A., Battaglia G., Floris M., Castellino P., Ronco C., Cruz D.N. (2011). Renal involvement in leishmaniasis: a review of the literature. NDT Plus.

[13] Hailu W., Mohamed R., Fikre H., Atnafu S., Tadesse A., Diro E. (2021). Acute kidney injury in patients with visceral leishmaniasis in Northwest Ethiopia. PLoS One.

[14] Daher E.F., da Silva Junior G.B., Trivedi M., Fayad T., Srisawat N., Nair S. (2022). Kidney complications of parasitic diseases. Nat Rev Nephrol.

[15] Abukhattab M., Daghfal J., Adam M., Hashim S.A., Mohammed F.K., Al-Maslamani M. (2024). Epidemiology, clinical characteristics, and treatment outcomes of leishmaniasis in Qatar: a retrospective study. Qatar Med J.

[16] Veroux M., Corona D., Giuffrida G., Cacopardo B., Sinagra N., Tallarita T. (2010). Visceral leishmaniasis in the early post-transplant period after kidney transplantation: clinical features and therapeutic management. Transpl Infect Dis.

[17] Hueso M., Bover J., Serón D., Gil-Vernet S., Rufí G., Alsina J. (1999). The renal transplant patient with visceral leishmaniasis who could not tolerate meglumine antimoniate-cure with ketoconazole and allopurinol. Nephrol Dial Transpl.

